# The RNA-Binding Protein RBM3 Promotes Neural Stem Cell (NSC) Proliferation Under Hypoxia

**DOI:** 10.3389/fcell.2019.00288

**Published:** 2019-11-21

**Authors:** Jingyi Yan, Tessa Goerne, Andrea Zelmer, Raphael Guzman, Josef P. Kapfhammer, Sven Wellmann, Xinzhou Zhu

**Affiliations:** ^1^Department of Neonatology, University Children’s Hospital Basel (UKBB), Basel, Switzerland; ^2^Department of Biomedicine, University of Basel, Basel, Switzerland; ^3^Department of Neonatology, University Children’s Hospital Regensburg (KUNO), Regensburg, Germany

**Keywords:** RBM3, CIRP, oxygen, neural stem cell, cell cycle

## Abstract

Neural stem cells (NSCs) reside physiologically in a hypoxic niche to maintain self-renewal and multipotency. Whereas mild hypoxia is known to promote NSC proliferation, severe hypoxia in pathological conditions exerts the reverse effect. The multi-functional RNA-binding protein RBM3 is abundant in NSCs and can be regulated by hypoxic exposure. Although RBM3 has been shown to accelerate cell growth in many cell types, whether and how it affects NSC proliferation in hypoxic environment remains largely unknown. In this study, we tested how RBM3 regulates cell proliferation under hypoxia in C17.2 mouse NSC cell line and in primary mouse NSCs from both the forebrain of postnatal day 0 (P0) mice and the subgranular zone (SGZ) of adult mice. Our results demonstrated that RBM3 expression was highly sensitive to hypoxia, and NSCs were arrested in G0/G1 phase by 5, 2.5, and 1% O_2_ treatment. When we overexpressed RBM3, hypoxia-induced cell cycle arrest in G0/G1 phase was relieved and more cell transit into S phase was observed. Furthermore, cell viability under hypoxia was also increased by RBM3. In contrast, in RBM3-depleted primary NSCs, less BrdU-incorporated cells were detected, indicating exacerbated cell cycle arrest in G1 to S phase transition. Instead, overexpressed RBM3 significantly increased proliferation ratio in primary NSCs. Our findings indicate RBM3 as a potential target to maintain the proliferation capacity of NSCs under hypoxia, which can be important in NSC-based therapies of acute brain injury and chronic neurodegenerative diseases.

## Introduction

Physiological oxygen levels in organisms are considerably lower than ambient oxygen tension (21%) and vary widely in different tissues to adapt oxygen consumption requirements of diverse cell types (Panchision, 2009; De Filippis and Delia, 2011). Neural stem cells (NSCs) are the main sources to generate neuronal and glial cells of the central nervous system during embryonic and postnatal development and are maintained in specific regions during adulthood for consistent neurogenesis (Gage and Temple, 2013; Bond et al., 2015). They also contribute to neuro-regeneration after acute injuries in the brain or spinal cord (Gage and Temple, 2013; Ludwig et al., 2018). Neurogenic niches, which are the microenvironment for NSC self-renewal and differentiation, contain a relatively wide range of oxygen tension from <1 to 8% (Mohyeldin et al., 2010). The heterogeneity of oxygen tension exerts opposite functions on NSC proliferation. While mild hypoxia substantially promotes NSC proliferation, severe hypoxia suppresses their growth and maintains them in quiescent status (Santilli et al., 2010; Felfly et al., 2011). Furthermore, different severity of hypoxia can also have opposite effects on NSC differentiation and neurogenesis (Francis and Wei, 2010; De Filippis and Delia, 2011; Vieira et al., 2011). Therefore, the precise control of hypoxic environment is critical for the maintenance of NSC quiescence/activation status and NSC amount in the pool, as well as for the regulation of their differentiation upon diverse demands.

The appropriate oxygen concentration can be different for *in vivo* animal studies and for *in vitro* cell models when investigating NSCs. Traditionally, 21% O_2_ is used as the standard laboratory oxygen supply concentration for cell culture (including NSC culture) *in vitro*, but some researchers questioned whether 21% O_2_ results in a relatively hyperoxic environment and may change the physiological characters of NSCs (Clarke and Van Der Kooy, 2009; Yuan et al., 2015; Mas-Bargues et al., 2019). Atmospheric oxygen tension *in vitro* may lead to a shift of NSC proliferation pattern. Therefore, lower oxygen level can be superior for NSC culture *in vitro*, when intending to mimic *in vivo* NSC characters. Instead, 8% O_2_ is considered as physiological oxygen tension in neurogenic niche, 2.5% O_2_ is considered as moderate hypoxia, and 1% O_2_ is considered as severe hypoxia (Panchision, 2009; De Filippis and Delia, 2011).

The multi-functional RNA-binding protein RBM3 is typically inducible by cold exposure (Danno et al., 2000; Zhu et al., 2016). Besides cold stress, RBM3 responds to hypoxia as well (Wellmann et al., 2004). During development, RBM3 expression is abundant in neurogenic niches and co-localizes with NSC marker nestin (Pilotte et al., 2009). RBM3 has been recently reported to promote neurogenesis via Yap during embryonic stage (Xia et al., 2018). Other studies also suggest that RBM3 plays an important role in the proliferation of cancer cells, fibroblasts, and HEK293 cells (Sureban et al., 2008; Wellmann et al., 2010; Matsuda et al., 2011; Chen et al., 2019). Besides, in recent years, a series of studies have demonstrated that RBM3 can promote the survival of neuroblastoma cells, which are widely used to replace NSCs in neuronal differentiation assays *in vitro*, upon diverse stressful treatments (Yang et al., 2017; Zhuang et al., 2017; Ushio and Eto, 2018). However, it remains unclear how RBM3 regulates NSC proliferation under hypoxic conditions.

In this study, we investigated whether RBM3 expression is affected under hypoxic conditions and elucidated the role of RBM3 in the regulation of cell cycle in mouse NSC cell line and primary murine NSCs exposed to hypoxia.

## Materials and Methods

### Animals

The research protocol, approved by the Cantonal Veterinary Office of Basel, was conducted according to the Ethical Principles and Guidelines for Experiments on Animals of the Swiss Academy of Medical Sciences and the Swiss Academy of Sciences. RBM3 wild type (WT) and knockout (KO) mice with C57BL/6 background were generated by Prof. Tadatsugu Taniguchi (University of Tokyo, Japan) (Matsuda et al., 2011) and were kindly provided by his group. Since *RBM3* is X-chromosome gene, only male mice were used in this study.

### Cell Isolation and Culture

Primary NSCs were isolated from the whole brain excluding cerebellum of postnatal day 0 (P0) mice or from the subgranular zone (SGZ) of 2-month-old adult mice as described previously (Zhu et al., 2019). Briefly, the forebrains from P0 mice or the dentate gyrus from adult mice were dissociated with papain (Worthington) and DNase I (Sigma) and then undissociated cell clusters were removed by a cell strainer (Sigma). Dissociated cells were cultured in serum-free DMEM-F12 (Gibco) supplemented with B27 supplement (Gibco), 2 mM L-glutamine (Gibco), 20 ng/ml EGF (PeproTech), and 20 ng/ml FGF2 (PeproTech). After glial cells and neurons died, primary NSCs were maintained as neurospheres in uncoated dishes.

C17.2 mouse NSC line was purchased from Sigma and cultured in DMEM (Gibco) supplemented with 10% FBS (Gibco) and 2 mM L-glutamine (Gibco).

### Plasmid Transient Transfection

pCEP4 mammalian expression vector was purchased from ThermoFisher Scientific. *rbm3* gene was cloned into pCEP4 vector in our previous work for exogenous overexpression (Chip et al., 2011). The empty vector or RBM3-overexpressing vector was transiently transfected into C17.2 cells by electroporation with Cell Line Nucleofector Kit V (Lonza) using the Nucleofector I device (Lonza). For transfections in primary NSCs, cells were first dissociated from neurospheres to single cells by Trypsin (Sigma) and then transfected with DNA vectors using the Mouse Neural Stem Cell Nucleofector Kit (Lonza) and the Nucleofector I device (Lonza).

### Hypoxia Exposure

Before hypoxic treatment, primary NSCs in the form of neurospheres were dissociated into single cells by Trypsin (Sigma) and seeded into poly-L-lysine pre-coated 16-well chamber slides (Labtek) at a density of 1 × 10^4^/well as monolayer culture in ambient normoxic condition (21% O_2_). For transfected primary NSCs, additional overnight recovery in uncoated 12-well plate was required before seeding to chamber slides. After 24 h (for non-transfected NSCs) or 48 h (for transfected NSCs) growth, 20 μM BrdU was added into the medium and the slides were transferred to a hypoxic incubator (MiniGalaxy A, RS Biotech) with indicated oxygen levels. Slides in an ambient normoxic incubator served as a control group.

Non-transfected and transfected C17.2 cells were seeded either in six-well plates or in 15-cm-diameter dishes, and transferred to a hypoxic incubator with indicated oxygen levels. Plates or dishes in ambient normoxic incubator served as a control group.

### RNA Isolation and Real-Time PCR

Total RNA were purified from 2 × 10^5^ cultured cells by the ReliaPrep RNA Cell Miniprep System (Promega). cDNA was synthesized from 1 μg total RNA using the GoScript Reverse Transcription System (Promega). Real-time PCR was performed in 15 μl volume with the GoTaq qPCR System (Promega) on the CFX Connect Real-Time PCR Detection System (Bio-Rad). The PCR cycle was run as follows: pre-denaturation by 95°C for 5 min, and then 95°C for 15 s and 60°C for 1 min for 40 cycles. To calculate relative gene expression, the 2^–ΔΔ*CT*^ method was used. Primer sequences for real-time PCR are listed below. To correct for differences in both RNA quality and quantity between samples as well as changes in oxygen exposure, six housekeeping genes were used, namely, beta-actin (*actb*), glyceraldehyde 3-phosphate dehydrogenase (*gapdh*), ribosomal protein L13a (*rpl13a*), 45S pre-ribosomal RNA (*rn45s*), 28S ribosomal RNA (*rn28s1*), and alpha-tubulin-1 (*tuba1*):

Mouse-*rbm3*-forward: 5′ CTT CAG CAG CTT TGG GCC TA 3′
Mouse-*rbm3*-reverse: 5′ CCC ATC CAG GGA CTC TCC AT 3′
Mouse-*cirp*-forward: 5′ CCA AGT ATG GGC AGA TCT CCG A 3′
Mouse-*cirp*-reverse: 5′ CTG CCG CCC GTC CAC AGA CT 3′
Mouse-*kdm3a*-forward: 5′ GAG CTG TTT CCC ACA CCG A 3′
Mouse- *kdm3a* -reverse: 5′ TGC TTT TCT CTG AAG GCT 3′
Mouse-*actb*-forward: 5′ GGC CAA CCG TGA AAA GAT GA 3′
Mouse-*actb*-reverse: 5′ CAC AGC CTG GAT GGC TAC GT 3′
Mouse-*gapdh*-forward: 5′ AAC GAC CCC TTC ATT GAC 3′
Mouse-*gapdh*-reverse: 5′ TCC ACG ACA TAC TCA GCA C 3′
Mouse-*rpl13a*-forward: 5′ GCG GAT GAA TAC CAA CCC 3′
Mouse-*rpl13a*-reverse: 5′ GTA GGC TTC AGC CGA ACA AC 3′
Mouse-*rn45s*-forward: 5′ GTA ACC CGT TGA ACC CCA TT 3′
Mouse-*rn45s-*reverse: 5′ CCA TCC AAT CGG TAG TAG CG 3′
Mouse-rn*28s1*-forward: 5′ TTG AAA ATC CGG GGG AGA G 3′
Mouse-*rn28s1*-reverse: 5′ ACA TTG TTC CAA CAT GCC AG 3′
Mouse-*tuba1*-forward: 5′ ACA GGA TTC GCA AGC TGG C 3′
Mouse-*tuba1*-reverse: 5′ CCA AGA AGC CCT GGA GAC C 3′

### Protein Isolation and Western Blot

Total proteins were extracted from cultured cells or homogenized mouse brain with lysis buffer (1% Triton X-100, 50 mM Tris, 150 mM NaCl, 1 × Roche Protease Inhibitor Cocktail, pH 8.0). Total protein concentrations in cleared cell lysates were determined with RC DC Protein Assay (Bio-Rad). Lysates were loaded onto Mini-Protean TGX pre-cast gels (Bio-Rad) and transferred to PVDF membranes (Amersham/GE Healthcare Life Sciences). Membranes were incubated with primary antibodies overnight at 4°C and then with HRP-linked secondary antibodies for 1 h at room temperature (RT).

Primary and secondary antibodies:

Anti-mouse RBM3 (Proteintech, 14363-1-AP): rabbit polyclonal, 1:1000 diluted
Anti-mouse CIRP (Proteintech, 10209-1-AP): rabbit polyclonal, 1:1000 diluted
Anti-mouse GAPDH (Abeam, ab8245): mouse monoclonal, 1:2000 diluted
HRP-linked anti-rabbit secondary antibody (Cell Signaling Technology, 7074S): 1:5000 diluted
HRP-linked anti-mouse secondary antibody (Cell Signaling Technology, 7076S): 1:5000 diluted.

Band relative quantification was performed by Image J (NIH). Each band was outlined and the mean grayscale value was measured. Background intensity was subtracted from the measured values. The ratios of target proteins (RBM3 and CIRP) and loading control (GAPDH) were calculated.

### Flow Cytometry

Flow cytometry analysis was performed with C17.2 cells on FACSCANTO II device (BD Biosciences). After hypoxic treatment, cells were fixed with ethanol for viability and cell cycle analysis. Cell viability was measured with 1 × 10^6^ cells using LIVE/DEAD Viability/Cytotoxicity Kit (ThermoFisher Scientific). For cell cycle analysis, 1 × 10^6^ cells were analyzed with propidium iodide (Sigma).

### Immunofluorescent Staining

After hypoxic treatment, cells in chamber slides were fixed 4% paraformaldehyde for 10 min at RT. For BrdU staining, fixed cells were treated 2 M HCl for 30 min and neutralized with 0.1 M sodium borate (pH 8.5) for 10 min before blocking. For permeabilization and blocking, cells were treated with 0.5% Triton X-100 and 5% normal goat serum in phosphate buffer for 1 h at RT. Cells were incubated with primary antibodies overnight at 4°C and then with Alexa Fluor dye-conjugated secondary antibodies for 2 h at RT. Nuclei were counterstained with DAPI for 15 min at RT. Images were acquired with an Olympus AX-70 fluorescent microscope equipped with a Spot Insight digital camera. For cell quantification, images of five random fields in each experiment were captured under 20 × magnifications, and the total numbers of BrdU + and DAPI + cells in each image were counted. The average percentage of BrdU + /DAPI + cells from five images was used to represent the value of one experiment. Three independent experiments were performed.

Primary antibodies:

Anti-Nestin (Novus, NBP1-02419), rabbit polyclonal, 1:200 diluted
Anti-Sox2 (R&D, MAB2018), mouse monoclonal, 1:250 diluted
Anti-Dcx (Millipore, AB2253), guinea pig polyclonal, 1:1000 diluted
Anti-Tuj1 (Tubulin III) (Millipore, AB1637), mouse monoclonal, 1:250 diluted
Anti-BrdU (Abcam, ab6326), rat monoclonal, 1:250 diluted.

### Statistical Analysis

All experiments were repeated three times independently. Quantification data are presented in standard error of the mean (SEM). Statistical analysis was performed by GraphPad Prism 8.0. To compare two groups with a single factor, unpaired *t* test was used. To compare more than two groups with a single factor, statistical significance was determined by one-way ANOVA followed by Dunnett’s multiple comparison. To compare two groups and two factors, statistical significance was determined by two-way ANOVA followed by Tukey’s multiple comparison. *p* value less than 0.05 was reported as significant difference. N.S., not significant; ^∗^*p* < 0.05; ^∗∗^*p* < 0.01.

## Results

### RBM3 Expression Is Sensitively Inhibited by Hypoxia in NSC

**FIGURE 1:**
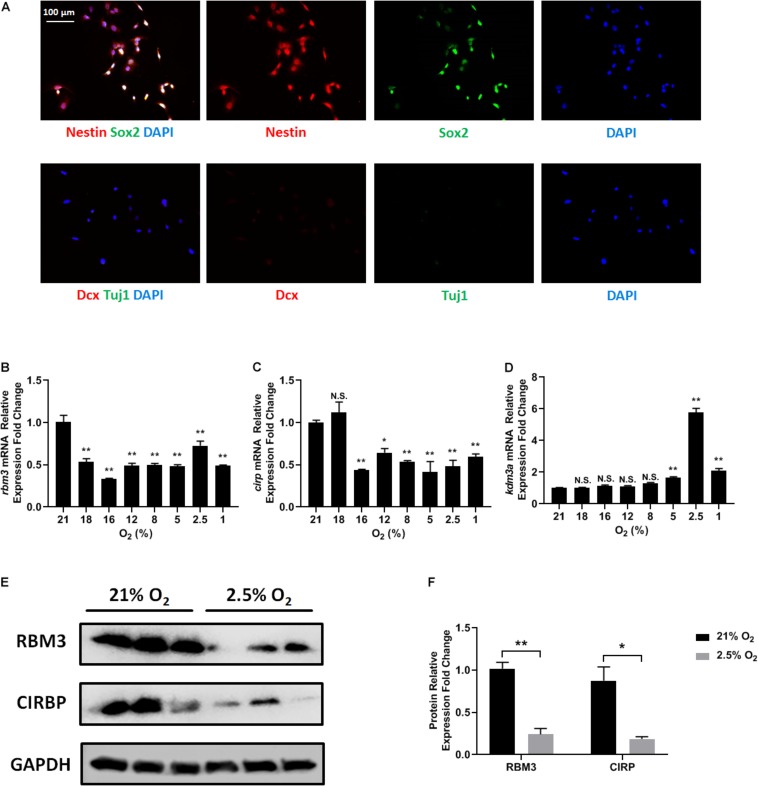
Oxygen-sensitive gene expression in NSCs upon hypoxic exposure. **(A)** Representative Nestin/Sox2 and Dcx/Tuj1 double stainings of C17.2 cells cultured in standard condition with 21% O_2_; nuclei were counterstained with DAPI. **(B–D)** mRNA expression of *rbm3* **(B)**, *cirp* **(C)**, and *kdm3a* **(D)** was measured 16 h after ambient normoxic (21% O_2_) or indicated hypoxic treatment. *Actb* was used as an internal control. One-way ANOVA followed by Dunnett’s test was used to compare each hypoxic condition to the normoxic group. N.S. not significant; ^∗^*p* < 0.05; ^∗∗^*p* < 0.01. **(E)** Representative Western blot, RBM3, and CIRP were measured 24 h after ambient normoxic (21% O_2_) or moderate hypoxic (2.5% O_2_) treatment; GAPDH was used as a loading control. **(F)** Band relative intensity in panel **(E)** was normalized, and unpaired *t* test was used for statistical analysis. N.S., not significant; ^∗^*p* < 0.05; ^∗∗^*p* < 0.01.

C17.2 mouse NSC line was used for this study, as it harbors NSC properties and is widely investigated with hypoxic treatments (Felfly et al., 2011; Wang et al., 2014; Zhang et al., 2017). We first confirmed the NSC property of C17.2 cells by immunostaining with specific markers. C17.2 cells express NSC marker nestin and sex determining region Y-box 2 (Sox2), but not neuroblast marker doublecortin (Dcx) and neuronal marker neuron-specific Class III β-tubulin (Tuj1) (Figure 1A), indicating their properties as undifferentiated NSCs.

To test how different oxygen levels influenced RBM3 expression, we exposed C17.2 cells to ambient normoxia (21% O_2_), very mild hypoxia (18 and 16% O_2_), mild hypoxia (12 and 8% O_2_), moderate hypoxia (5 and 2.5% O_2_), and severe hypoxia (1% O_2_). Beta-actin gene (*actb*) was selected as reference gene for real-time PCR, as it showed the highest stability under hypoxic conditions among six commonly used housekeeping genes (Supplementary Table S1). The mRNA level of *rbm3* was remarkably suppressed even under very mild hypoxic condition, and remained suppressed with mild, moderate, and severe hypoxia (Figure 1B). Notably, at 2.5% O_2_, *rbm3* expression was recovered to some extent, but remained lower than ambient normoxic condition (Figure 1B). *kdm3a*, a well-characterized hypoxia-inducible gene, was used as positive control (Wellmann et al., 2008), and its expression was upregulated by moderate to severe hypoxia (Figure 1D). At 2.5% O_2_ level, *kdm3a* expression reached the peak (Figure 1D). Interestingly, the only known vertebrate homolog of RBM3, cold inducible RNA-binding protein (CIRP) (Zhu et al., 2016), showed a similar reduction of mRNA as *rbm3* under hypoxic conditions, but not as sensitive as *rbm3* at 18% O_2_ level (Figure 1C). Consistently, RBM3 and CIRP protein expressions were also downregulated by 2.5% hypoxia (Figures 1E,F). In general, our results show that RBM3 expression is extremely sensitive to hypoxic exposure in C17.2 mouse NSC line.

### Moderate to Severe Hypoxia Inhibits C17.2 Cell Proliferation

**FIGURE 2:**
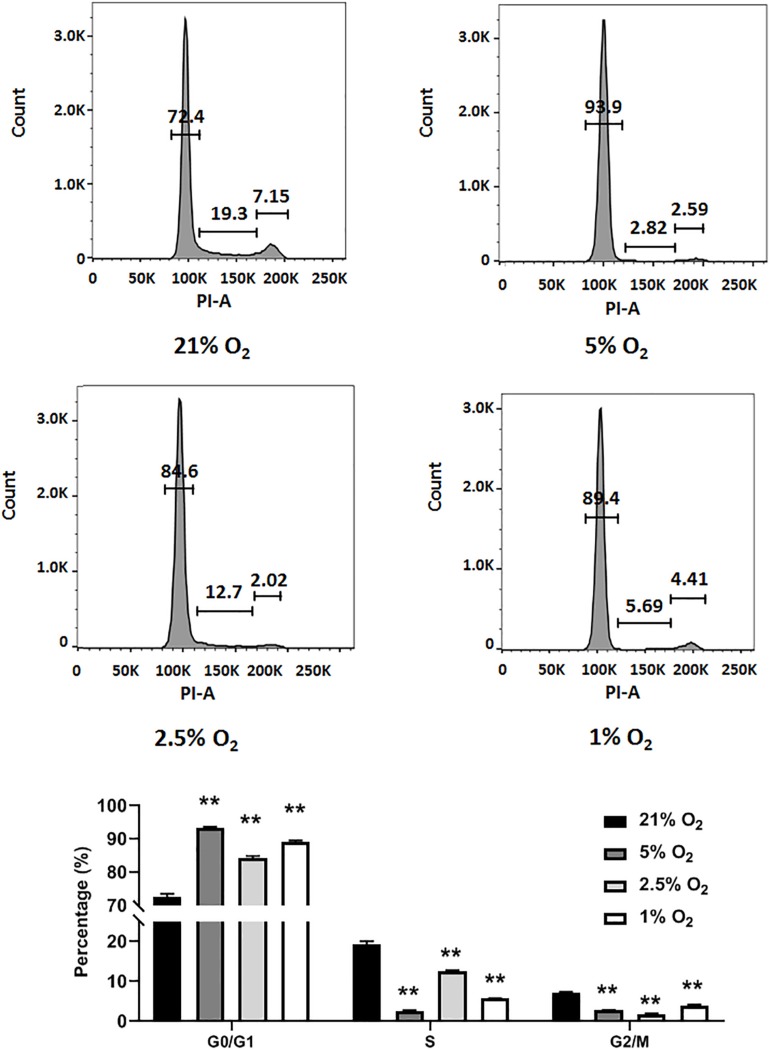
Hypoxia induces cell cycle arrest in NSCs. Cell cycle analysis of C17.2 cells after 24 h 5% O_2_, 2.5% O_2_, and 1% O_2_ hypoxic treatment. Ambient normoxic (21% O_2_) was used as control. Representative flow cytometry DNA histogram with propidium iodide (PI) staining. One-way ANOVA followed by Dunnett’s test was used for statistical analysis in G0/G1 phase, S phase, and G2/M phase (*n* = 3). N.S., not significant; ^∗∗^*p* < 0.01.

In general, NSCs are exposed to moderate hypoxia physiologically and to severe hypoxia pathologically *in vivo* (De Filippis and Delia, 2011). Here, we evaluated how different degrees of moderate to severe hypoxia influenced the cell cycle of C17.2 cells. Cells were treated with 21, 5, 2.5, and 1% O_2_ for 24 h, and then stained with propidium iodide and analyzed for cell cycle change by flow cytometry. We observed that moderate (5 and 2.5% O_2_) and severe hypoxia (1% O_2_) lead to a remarkable cell cycle arrest, with increased cell number in G0/G1 phase and decreased cell number in S phase (Figure 2). Cell numbers in S phase and G2/M phase were reduced in all hypoxic groups (Figure 2). In accordance to a previous report, our data supported the idea that severe hypoxia (1% O_2_) inhibited cell cycle in C17.2 cells (Zhang et al., 2017). However, with moderate hypoxia (5 and 2.5% O_2_), we observed an inhibitory effect as well (Figure 2) rather than a stimulating effect on NSC proliferation (Zhang et al., 2017). As moderate hypoxia (2.5% O_2_) demonstrated more cell arrest in S phase than 5 and 1% hypoxia (Figure 2), and a recovery of expression was observed (Figure 1B), in the following RBM3-overexpressing experiments, we selected this oxygen concentration to examine the function of RBM3 on NSC proliferation and survival.

### Overexpression of RBM3 Rescues C17.2 Cell From Hypoxia-Induced Cell Cycle Arrest

**FIGURE 3:**
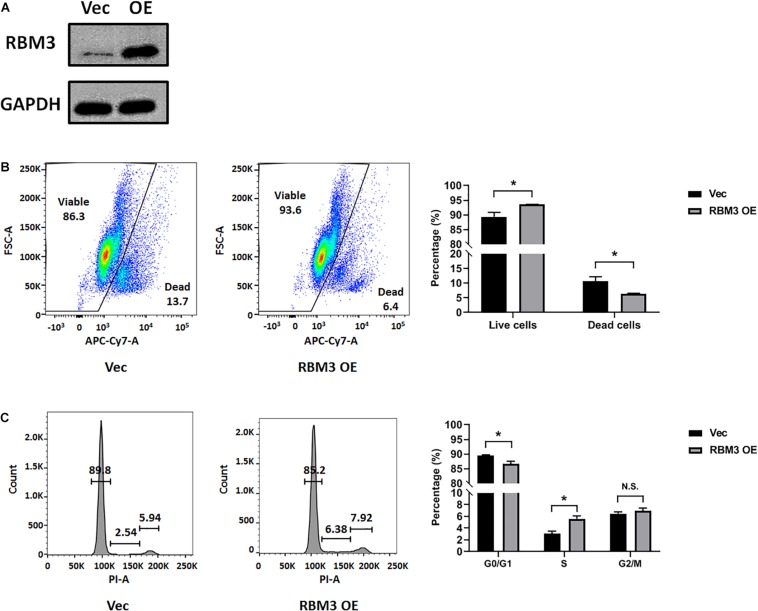
RBM3 overexpression increases NSC viability and proliferation under moderate hypoxia. **(A)** RBM3 protein expression after transient transfection with overexpressing vector. Vec: empty vector; RBM3 OE: RBM3-overexpressing vector. **(B)** Representative flow cytometry dot plots of transfected C17.2 cells with LIVE/DEAD viability staining after 24 h moderate hypoxia (2.5% O_2_). Unpaired *t* test was used for statistical analysis (*n* = 3). ^∗^*p* < 0.05. **(C)** Representative flow cytometry DNA histogram of transfected C17.2 cells with propidium iodide (PI) staining after 24 h moderate hypoxic (2.5% O_2_) treatment. Vec: empty vector; RBM3 OE: RBM3-overexpressing vector. Unpaired *t* test was used for statistical analysis in G0/G1 phase, S phase and G2/M phase (*n* = 3). N.S., not significant; ^∗^*p* < 0.05.

To explore the role of RBM3 in hypoxia-induced cell cycle arrest, we overexpressed RBM3 in C17.2 cells by transient transfection (Figure 3A) before exposing them to moderate hypoxia (2.5% O_2_). As RBM3 was found to be cytoprotective (Yang et al., 2017; Zhuang et al., 2017; Ushio and Eto, 2018), we examined the viability of NSCs after moderate hypoxic treatment. Forced RBM3 expression significantly enhanced live cell proportion and reduced dead cell proportion (Figure 3B). While cells were arrested in G0/G1 phase after hypoxic treatment, RBM3 overexpression caused the cells to overcome the arrest (Figure 3C). These data provide evidence that RBM3 positively regulates NSC growth under moderate hypoxic condition by promoting G1 to S phase transition.

### RBM3 Positively Regulates Primary NSC Proliferation Under Hypoxia

**FIGURE 4:**
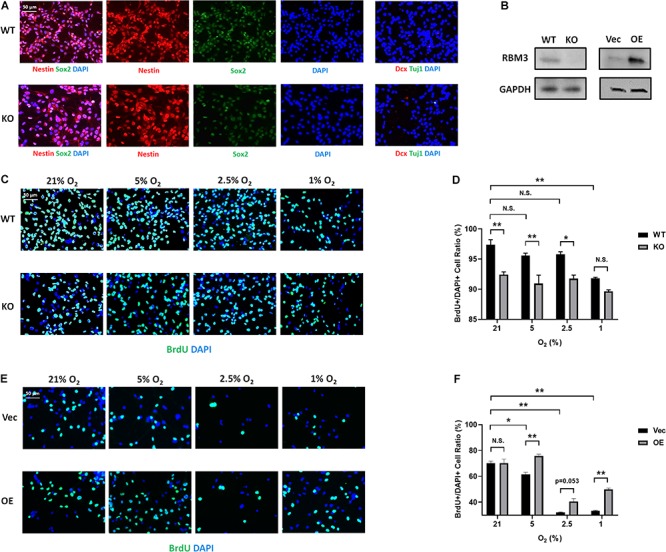
RBM3 positively regulates proliferation in primary NSC from P0 mice under hypoxia. **(A)** Representative Nestin/Sox2 and Dcx/Tuj1 double staining of primary RBM3 WT and KO NSCs from the forebrains of P0 mice. Nuclei were counterstained with DAPI. **(B)** RBM3 protein expression in RBM3 WT and KO primary NSCs, and vector transfected WT NSCs from the forebrains of P0 mice. Vec: empty vector; OE: RBM3-overexpressing vector. **(C)** Representative BrdU and DAPI staining of P0 WT or KO NSCs after 24 h 21, 5, 2.5, or 1% O_2_ treatment. **(D)** Two-way ANOVA followed by Tukey’s test was used for the comparisons in panel **(C)**. N.S., not significant; ^∗^*p* < 0.05; ^∗∗^*p* < 0.01. **(E)** Representative BrdU and DAPI staining of P0 WT NSCs transfected with empty or RBM3-overexpressing vector after 24 h 21, 5, 2.5, or 1% O_2_ treatment. **(F)** Two-way ANOVA followed by Tukey’s test was used for the comparisons in panel **(E)**. N.S., not significant; ^∗^*p* < 0.05; ^∗∗^*p* < 0.01.

To further confirm the role of RBM3, we took advantage of primary NSCs isolated from the forebrain of postnatal day 0 (P0) RBM3 WT and KO mice. Similar to C17.2 cells, both WT and KO NSCs express NSC marker nestin and Sox2, but neither the neuroblast marker Dcx nor the neuronal marker Tuj1 (Figure 4A). RBM3 depletion in KO NSCs was confirmed by Western blot (Figure 4B). We further treated primary NSCs with ambient normoxia (21% O_2_), moderate hypoxia (5 and 2.5% O_2_), or severe hypoxia (1% O_2_), and labeled cells by BrdU, a commonly used proliferation marker for S phase (Lengronne et al., 2001). After 24 h incorporation with BrdU, a significant decrease of proliferation ratio (% of BrdU + /DAPI + cells) from ambient normoxia (21% O_2_) to severe hypoxia (1% O_2_) was detected, but not from ambient normoxia (21% O_2_) to moderate hypoxia (5 and 2.5% O_2_) (Figures 4C,D), probably due to the robustness of P0 NSCs with high proliferation rate and resistance to hypoxic stress. The proliferation ratio was remarkably downregulated in KO NSCs when comparing to WT NSCs under ambient normoxic condition (21% O_2_) or moderate hypoxic conditions (5 and 2.5% O_2_), but not significant under severe hypoxic conditions (1% O_2_) (Figures 4C,D). In addition, we overexpressed exogenous RBM3 in primary P0 NSCs by electroporation (Figure 4B). Electroporation-based transfection significantly reduced proliferation ratio to around 70% under normoxic conditions (21% O_2_), while both moderate hypoxia (5 and 2.5% O_2_) and severe hypoxia (1% O_2_) further inhibited proliferation ratio remarkably (Figures 4E,F). In contrast to depletion, forced RBM3 expression rescued hypoxia-induced proliferation inhibition partially, but did not alter proliferation under normoxia (21% O_2_) (Figures 4E,F).

**FIGURE 5:**
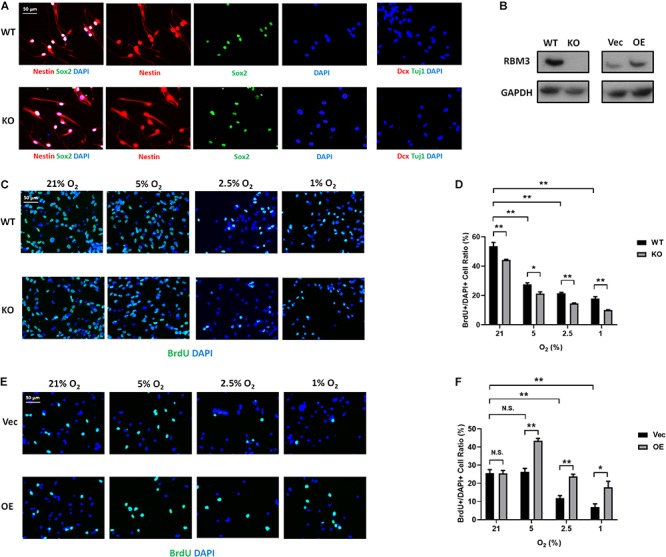
RBM3 positively regulates proliferation in primary NSC from the SGZ of adult mice under hypoxia. **(A)** Representative Nestin/Sox2 and Dcx/Tuj1 double staining of primary RBM3 WT and KO NSCs from the SGZ of adult mice. Nuclei were counterstained with DAPI. **(B)** RBM3 protein expression in RBM3 WT and KO primary NSCs, and vector transfected WT NSCs from the SGZ of adult mice. Vec: empty vector; OE: RBM3-overexpressing vector. **(C)** Representative BrdU and DAPI staining of SGZ-derived adult WT or KO NSCs after 24 h 21, 5, 2.5, or 1% O_2_ treatment. **(D)** Two-way ANOVA followed by Tukey’s test was used for the comparisons in panel **(C)**. ^∗^*p* < 0.05; ^∗∗^*p* < 0.01. **(E)** Representative BrdU and DAPI staining of SGZ-derived adult WT NSCs transfected with empty or RBM3-overexpressing vector after 24 h 21, 5, 2.5, or 1% O_2_ treatment. **(F)** Two-way ANOVA followed by Tukey’s test was used for the comparisons in panel **(E)**. N.S., not significant; ^∗^*p* < 0.05; ^∗∗^*p* < 0.01.

To confirm that the effect of RBM3 in NSC proliferation is general, we isolated NSCs from the SGZ of adult mice, one of the two well-characterized neurogenic niches in adults (Fuentealba et al., 2012). We did not use subventricular zone (SVZ)-derived NSCs because our previous study showed that RBM3 plays a less important role in the proliferation of SVZ-NSCs than SGZ-NSCs after hypoxic–ischemic (HI) injury (Zhu et al., 2019). The characters of SGZ-derived NSCs were confirmed by positive staining of nestin/Sox2 and negative staining of Dcx/Tuj1 (Figure 5A). We also confirmed RBM3 depletion in KO SGZ-NSCs and RBM3 overexpression in electroporation-transfected WT SGZ-NSCs (Figure 5B). We also observed a reduction of proliferation of SGZ-NSC in the absence of RBM3 under hypoxia (Figures 5C,D). On the other hand, overexpressed RBM3 elevated SGZ-NSC proliferation ratio under hypoxic conditions (Figures 5E,F).

## Discussion

In this study, we exposed murine NSCs to various degrees of hypoxia and noticed a remarkable downregulation of RBM3 even at very mild hypoxia (18% O_2_) with little further change at lower oxygen levels (Figure 1B). The sensitivity of RBM3 expression to hypoxic response is higher than its homolog CIRP (Figures 1B,C). Such a high sensitivity of RBM3 has also been reported in response to environmental temperature change, more than CIRP. Even a 1°C change from 37 to 36°C is sufficient to stimulate RBM3 expression in primary cortical neurons and astrocytes (Jackson et al., 2015). Regarding hypoxia, CIRP expression was also reported to decrease in moderate (3%) and severe (1%) hypoxic conditions (Zhang et al., 2017). However, RBM3 was previously found to be induced by moderate (5%) and severe (1%) hypoxia in HeLa and Hep3B cancer cells (Wellmann et al., 2004). The discrepancy in different cell types indicates that the subtle regulation patterns of the two cold-inducible RNA-binding proteins RBM3 and CIRP by hypoxia can be cell type specific and may involve different regulatory mechanisms. Indeed, cancer stem cells reside in a more hypoxic niche than NSCs and utilize oxygen in different signaling pathways compared to NSCs, in the maintenance of their stemness (Panchision, 2009; Mohyeldin et al., 2010). Notably, in C17.2 cell line, both RBM3 and CIRP are downregulated under moderate to severe hypoxic conditions (Figures 1B,C; Zhang et al., 2017), and both proteins promote G1/S transition in the cell cycle of NSCs upon hypoxic exposure (Figure 3C; Zhang et al., 2017), in accordance with their homogeneous structure and functions as reviewed before (Zhu et al., 2016). We additionally demonstrated consistent effects of RBM3 on NSC proliferation in neonatal and adult primary NSCs (Figures 4, 5). Based on these functions, both RBM3 and CIRP are suggested for potential prevention of hypoxia-induced brain injury. However, considering that CIRP is also involved in mediating neuro-inflammation (Rajayer et al., 2013; Zhou et al., 2014), instead, RBM3 tends to be a safer target in clinical use.

Ischemic stroke and global HI injury are the most common cerebral hypoxic injuries, which induce irreversible damage to the brain from infants to adults (Huang and Castillo, 2008; Ekker et al., 2018). Although NSCs normally reside in hypoxic niche in physiological conditions, they can also migrate to injured regions for neuro-regeneration in these pathological conditions with moderate to severe hypoxia. Our recent study provides evidence that RBM3 behaves as a potential target to maintain NSC pool in HI conditions (Zhu et al., 2019). In addition, as the endogenous migrating NSCs are usually not sufficient to replace lost neurons by neurogenesis, exogenous transplantation of NSC is required for improved recovery not only by cell replacement, but also by multiple by-stand mechanisms (Kalladka and Muir, 2014; Vishwakarma et al., 2014; Huang and Zhang, 2019). When preparing exogenous NSCs *in vitro*, physiologically hypoxic pre-conditioning may benefit to mimic *in vivo* environment (Wakai et al., 2016), but can also produce disadvantageous effects, as hypoxia is a complex process. As the promotion of proliferation is largely mediated by hypoxia-inducible factor-1α (HIF-1α) signaling (Mohyeldin et al., 2010; Li et al., 2014), RBM3 regulation is independent of HIF-1α (Wellmann et al., 2004). Therefore, manipulating RBM3 expression opens a new avenue to increase viability, proliferation capacity, and multi-potency of cultured NSCs and probably other types of stem cells, which may improve therapeutic effects after transplantation.

## Data Availability Statement

All datasets generated for this study are available upon request to the corresponding author.

## Ethics Statement

The animal experiment involved in this study was approved by the Cantonal Veterinary Office of Basel (License Number 2064). All the manipulations were executed according to the Ethical Principles and Guidelines for Experiments on Animals of the Swiss Academy of Medical Sciences and the Swiss Academy of Sciences.

## Author Contributions

XZ and SW designed the study, interpreted data, and wrote the manuscript. JY and TG performed all experiments in part assisted by AZ. RG and JK were involved in the interpretation and discussion of the results. All authors approved the manuscript.

## Conflict of Interest

The authors declare that the research was conducted in the absence of any commercial or financial relationships that could be construed as a potential conflict of interest.

## Supplementary Material

TABLE S1 Stability test of housekeeping genes under hypoxia for real-time PCR. The *Ct* values of six commonly used housekeeping genes, beta-actin (*actb*), glyceraldehyde 3-phosphate dehydrogenase (*gapdh*), ribosomal protein L13a (*rpl13a*), 45S pre-ribosomal RNA (*rn45s*), 28S ribosomal RNA (*rn28s1*) and alpha-tubulin-1 (*tuba1*), were measured by real-time PCR under 21, 5, 2.5, and 1% hypoxic conditions in C17.2 cells. Their stability was ranked by geNorm method (https://genorm.cmgg.be/). *Actb* shows the lowest *M* value, indicating its highest stability under hypoxic conditions and thereby selected as reference gene in real-time PCR experiments.
